# Evaluating In Vitro Performance of a Novel Stainless Steel Rotary System (Gentlefile) Based on Debris Extrusion and Instrumentation Time

**DOI:** 10.1155/2023/9945236

**Published:** 2023-10-30

**Authors:** Ahmad Nouroloyouni, Fatemeh Safavi Hir, Robab Farhang, Sara Noorolouny, Amin Salem Milani, Rashin Alyali

**Affiliations:** ^1^Department of Endodontics, School of Dentistry, Ardabil University of Medical Science, Ardabil, Iran; ^2^Department of Pediatric Dentistry, School of Dentistry, Ardabil University of Medical Science, Ardabil, Iran; ^3^Department of Endodontics, School of Dentistry, Tabriz University of Medical Science, Tabriz, Iran; ^4^Department of Dentistry, Guilan University of Medical Sciences, Rasht, Iran

## Abstract

The new Gentlefile (GF) system, made of stainless steel and developed by MedicNRG in Kibbutz Afikim, Israel, claims to have advantages over traditional nickel-titanium files. However, research has shown that nickel-titanium files are mechanically superior due to their increased flexibility, cutting efficiency, and ability to maintain canal anatomy with less risk of procedural errors. This study compared the amount of debris extrusion and the time required for root canal instrumentation using GF versus the nickel-titanium ProTaper Universal (PTU) system and a manual step-back (MSB) stainless steel technique. This in vitro experimental study utilized 66 extracted human single-canal mandibular premolars with mature apices and root curvature of less than 10 degrees. The teeth were randomly divided into three groups (*n* = 22) and standardized for working length before being placed in preweighed vials. Group 1 was instrumented with PTU, Group 2 with GF, and Group 3 with the MSB technique. Extruded debris was collected in the vials, dried in an incubator, and weighed using the same scale. The change in weight indicates the debris amount. Instrumentation time was recorded using a stopwatch. The normal distribution of data was assessed using the Kolmogorov-Smirnov test. The groups were then compared regarding the amount of extruded debris and instrumentation time using the Kruskal-Wallis test and one-way ANOVA, followed by the Games-Howell test, respectively (alpha = 0.05). No significant difference in apical debris extrusion was found among the three groups (*P* > 0.05). However, a significant difference in instrumentation time was detected between the groups (*P* < 0.05). MSB instrumentation took significantly longer than both the PTU (*P* = 0.001) and GF (*P* = 0.001) systems. The GF system did not demonstrate reduced apical debris extrusion or faster instrumentation time compared to PTU. MSB had the longest instrumentation time compared to the other techniques.

## 1. Introduction

Chemomechanical root canal instrumentation is often associated with the extrusion of necrotic debris, residual pulp tissue, microorganisms, and irrigants into the periapical tissue [1]. Apical extrusion of debris can elicit a severe inflammatory response and may be clinically associated with pain and edema [2, 3]. Thus, minimizing the apical extrusion of debris should be prioritized by selecting the most suitable instrumentation technique. In other words, a high amount of extruded debris is often the result of inappropriate mechanical instrumentation of the root canal system [4].

Apical extrusion of debris following root canal instrumentation with various techniques and instruments has been the subject of numerous investigations [5]. The available literature indicates that all commonly adopted instrumentation techniques cause apical extrusion of debris. However, the amount of extruded debris varies among different systems and file designs [6]. Moreover, it has been documented that a lower extrusion of debris is often associated with a better treatment outcome [7].

Gentlefile (GF) (MedicNRG, Kibbutz Afikim, Israel) introduces a novel approach to endodontic procedures using stainless steel [8]. This system's unique design incorporates specialized files with a multipart structure. In the apical third of the file, there is a central braided cable that is less than 0.15 mm wide. This cable has a second smaller wire, measuring under 0.20 mm, wrapped around it. Moving towards the middle and coronal regions of the instrument, a third wire no greater than 0.35 mm coils over the second.

The last 0.5 mm of the apical end is sharpened at a 45-degree angle to create a passive, noncutting tip. The files have a consistent 4% taper and an inactive, passive tip. Notably, the tip diameters of 0.21, 0.23, 0.26, 0.29, and 0.34 mm deviate from the standard ISO dimensions for endodontic instruments [8].

The manufacturer claims that the GF has been designed to minimize pressure and maximize cleaning efficiency. The unique design of this product is intended to minimize the unnecessary removal of tooth structure, while still allowing for effective cleaning of the root canal system and preservation of the original root canal anatomy. The GF files are composed of stainless steel and exhibit exceptional flexibility as a result of their distinctive design, rendering them remarkably resistant to fractures [8].

The GF instrument demonstrated reduced apical transportation within the 5–7 mm segment, in contrast to ProTaper Next. Additionally, the GF instrument surpassed other techniques in efficiently removing smear layers. The coarse external texture of GF files facilitates the even expulsion of debris from the root canal, ensuring consistent and uniform shaping of the root canal walls [9].

The GF files rotate at a speed of 6500 rpm and have a nearly negligible torque due to their specific material composition and design. This characteristic effectively safeguards against root canal deformation. Additionally, this system features a specific portable hand-piece with adjustable angulation, providing the files with high flexibility. This system has six files of different colors and sizes: gray (coronal file, 20 mm, 022 tip), black (25 mm, 034 tip), green (25 mm, 029 tip), blue (25 mm, 026 tip), red (25 mm, 023 tip), and yellow (25 mm, 021 tip) [5–7]. To the best of the authors' knowledge, there is very limited information regarding the extrusion of debris following the use of GF files [8, 9].

ProTaper Universal (PTU) files are made of conventional nickel-titanium alloys. They have a convex triangular cross-sectional design, a safe noncutting tip, and a flute design with a variable taper [10]. Instruments with this specific cross-sectional design have higher dentin-cutting efficiency [11]. PTU is often used as a reference for the purpose of comparing with other files regarding apical extrusion of debris [12, 13]. PTU often shows minimal extrusion of debris [14].

The step-back technique enlarges the canal in a graduated manner by using sequentially larger files. A recent randomized controlled trial found that patients experienced less intense pain with the manual step-back approach versus other common techniques such as crown-down, hybrid, and conventional instrumentation [15]. This suggests that the step-back technique may result in less debris extrusion.

A recent randomized controlled single-blind clinical trial also suggested that longer instrumentation times could lead to increased damage to the tissues surrounding the tooth apex. This could result in an increase in postoperative pain. Therefore, assessing the duration of instrumentation appears beneficial for reducing posttreatment discomfort [16].

Considering the differences in instrument designs and applications for root canal treatment, there may be variations in the extrusion of debris. Concerning the clinically adverse effects of apical extrusion of debris, this study was aimed at comparing the amount of debris extruded apically and the time required for root canal instrumentation using the GF system versus the PTU and MSB technique. The null hypothesis was that no significant difference would be found in the amount of apically extruded debris and instrumentation time among the three groups.

## 2. Materials and Methods

This in vitro experimental study utilized 66 extracted human mandibular first and second premolars with single canals. The teeth were extracted for orthodontic treatment or advanced periodontal disease. The study protocol was approved by the Research Ethics Committee of Ardabil University of Medical Sciences (IR.ARUMS.REC.1398.226). Sample size was calculated to be 22 teeth per group based on a previous study by Nevares et al. [17] with an alpha of 0.05, beta of 0.2, and power of 80% using PASS 11 software (NCSS, Kaysville, Utah, USA).

The inclusion criteria were single-rooted, single-canal extracted human mandibular premolars. The exclusion criteria included the presence of more than one apical foramen, teeth with internal or external root resorption, calcification, previous endodontic treatment, immature root apex, root fracture or cracks, or a canal curvature greater than 10 degrees according to Schneider's classification [18]. Additionally, teeth with an initial file size larger than the #20 K-file were also excluded.

The collected teeth were radiographed from the buccal and proximal directions to ensure that they met the eligibility criteria. They were then immersed in a 0.5% chloramine solution (Merck, Darmstadt, Germany) for 48 hours for disinfection. They were stored in distilled water at 4°C until they were used. The root surfaces were debrided using a Cavitron ultrasonic scaler and cleaned with a prophy brush. Straight-line access was established in all teeth. Also, the cusp tip was flattened in all teeth in order to standardize the root length. To calculate the working length, a #15 K-file was inserted into the canal until its tip was visible at the apex. The working length was then determined to be 1 mm shorter than the canal length. To quantify the amount of extruded debris during root canal instrumentation, we adopted the technique by Myers and Montgomery [19].

**Figure 1:**
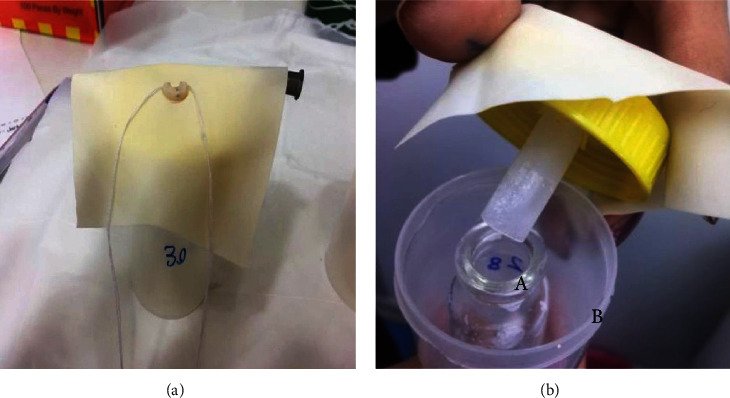
(a) Collection of apically extruded debris using the Myers and Montgomery method in which the tooth was inserted through the stopper of Eppendorf tubes. (b) A: collective vial; B: flask.

For this purpose, the teeth were inserted through the stopper of Eppendorf tubes, the end of the tubes was trimmed, and the assembly was secured in the rubber cap of a larger vial. The gaps around the stopper were filled with glue to prevent any leakage. The root end was inserted into a smaller vial, which was then placed inside the larger flask (Figure 1). The small vials were labeled and weighed three times using a digital scale with 0.01 mg accuracy (CPA225D, Sartorius, Germany). A 27-gauge needle was used to equalize the air pressure inside and outside of the vial. A rubber dam was then placed on the teeth to ensure that the operator was blinded. The teeth were then randomly divided into three groups (*n* = 22) as follows:

*Group 1*: root canal instrumentation in this group was performed using stainless steel hand K-files with a length of 21 mm and a taper of 0.02 (Mani, Tohnichi, Japan) with a quarter-pull motion and the step-back technique. The apical part was prepared up to the #25 K-file.

*Group 2*: root canal instrumentation was performed using SX, S1, S2, F1, and F2 files from PTU (Dentsply Maillefer, Ballaigues, Switzerland). An electric motor (Endo E Class, Saeyang, Marathon, Korea) was used for full-rotation movements. As per the manufacturer's guidelines, the speed was 300 rpm and the torque for the Sx, S1, and S2 shaping files was 1.5 Ncm, and for the F1 and F2 finishing files, it was 3 Ncm.

*Group 3*: the GF rotary system (Gentlefile; MedicNRG, Kibbutz Afikim, Israel) with a 4% taper was used to prepare the apical region. The preparation was done in an orderly manner using the following tips: #gray 022 tip, #black 034 tip, #green 029 tip, #blue 026 tip, and #red 023 tip. The GF files rotate at a speed of 6500 rpm in their specific handpiece and have a nearly negligible torque due to their specific material composition and design. A picking motion with direct apical pressure was applied for 5 seconds.

EDTA solution was employed as a lubricant for all instruments. In each experimental group, a #10 K-file was systematically inserted into the root canal following the use of each instrument to verify and maintain its patency. Additionally, following the utilization of each instrument and the execution of three up-and-down movements with rotary files, the root canals were rinsed with 2 cc of 5.25% sodium hypochlorite. This was accomplished by employing a side-vented 27-gauge needle, which was inserted 1 mm short of the working length. The debris and irrigant that were extruded coronally were removed using suction. In order to ensure consistency in the size of the apical preparation, #25 master apical files were utilized across all groups. Debris that had accumulated in the vial, as well as the debris that had adhered to the apex, were both collected by rinsing the apex with 1 cc of distilled water. Next, all vials containing debris and distilled water were subjected to incubation at a temperature of 70°C for a duration of 5 days in order to promote desiccation. The vials were subsequently weighed three times utilizing a consistent digital scale with a precision of 0.01 mg. The calculation of the mean of the values was subsequently performed. The quantification of apically extruded debris involved the determination of weight difference between the initial and final measurements of the vials. The duration of the preparation process was measured in seconds using a chronometer.

### 2.1. Statistical Analysis

The normal distribution of data was assessed using the Kolmogorov-Smirnov test. The groups were then compared regarding the amount of extruded debris and instrumentation time using the Kruskal-Wallis test for nonparametric data (amount of extruded debris) and one-way ANOVA, followed by the Games-Howell test for parametric data (instrumentation time). The analysis was conducted using SPSS version 25 (SPSS Inc., Chicago, IL, USA). A *P* value of less than 0.05 was considered statistically significant.

## 3. Results

### 3.1. Debris Extrusion

The results of the Kolmogorov-Smirnov test revealed that the distribution of the debris extrusion variable is not normal.

The results of the Kruskal-Wallis test are presented in Table 1.

### 3.2. Time

The mean and standard deviation of time in the studied groups are presented in Table 2.

The results of the Kolmogorov-Smirnov test suggest that the distribution of the time variable follows a normal distribution. The one-way ANOVA test was employed, to analyse disparity in the average time among the groups under investigation. The results are present in Table 2.

To determine the groups between which this difference exists, the Games-Howell test was employed. The findings showed the following:

1. There was a statistically significant difference in mean time between the ProTaper and Gentlefile groups, with the mean time lower in the ProTaper group (*P* value < 0.05)
2. There was a statistically significant difference in the mean time between the ProTaper and Manual groups, with the mean time lower in the ProTaper group (*P* value < 0.001)
3. There was a statistically significant difference in the mean time between the Gentlefile and the manual groups, with the mean time lower in the Gentlefile group (*P* value < 0.001)

## 4. Discussion

This study was aimed at comparing the extent of apical debris extrusion and the time required for instrumentation when preparing root canals using three different files: the GF system, PTU, and MSB. The null hypothesis was that no significant difference would be found in the amount of apically extruded debris and instrumentation time among the three groups. The study was conducted in vitro. PTU was used as the established benchmark in this study. The evaluation of the GF system was conducted in response to claims that this stainless steel system offers advantages over traditional nickel-titanium files. Additionally, a recent randomized controlled trial discovered that patients reported a reduction in pain intensity when the manual step-back approach was utilized, as compared to other commonly employed techniques such as crown-down, hybrid, and conventional instrumentation [15]. The aforementioned observation implies that the utilization of the step-back technique could potentially lead to a reduction in the extrusion of debris.

The findings indicated that there was no statistically significant variation in the extrusion of apical debris among the three experimental groups. Thus, the study's null hypothesis regarding debris extrusion was accepted by the findings. However, PTU and GF were significantly faster than MSB. Thus, the null hypothesis of the study about the instrumentation time was refused. Apical extrusion of debris is an inherent side effect during the process of root canal preparation. All the instrumentation techniques were found to be associated with apical debris extrusion in this study, which aligns with previous investigations [20–22]. The quantity of extruded debris is subject to variation depending on the design and kinematics of the instrument [5, 6]. In the current study, Gentlefile did not demonstrate reduced debris extrusion when compared to ProTaper Universal or manual instrumentation. The triple-wire design and high flexibility of Gentlefile files with improved cleaning ability [5, 23] were expected to minimize extrusion. However, the findings suggest that the physical properties of the files were not effective in adequately reducing apical extrusion.

A recent randomized clinical trial has suggested that when the same clinician performed root canal treatment on similar teeth using two different techniques with the same file system, there was no statistically significant difference in postoperative pain [24]. Additionally, the current study found no significant differences in apical debris extrusion among the three groups. These results further emphasize that postoperative pain is influenced by many factors, including the condition of the tooth and the expertise of the clinician carrying out the procedure.

In contrast to the current findings, previous studies conducted by Preethy et al. [25] and Zarrabi et al. [26] reported a higher extrusion of debris during manual instrumentation compared to rotary instruments. The present study underscores the multifactorial nature of debris extrusion and suggests that variations in file design alone may not have a substantial effect on this outcome.

In single-canal teeth, it has been reported that there is a lower apical extrusion of debris. This can be attributed to larger root canal diameter and the reduced piston-like effect of the files [27]. Accordingly, a smaller diameter of the root canal, such as in the mesial canal of mandibular molars, has been found to be associated with a greater amount of debris being extruded apically. In the current investigation, the working length was established to be 1 mm shorter than that of the apical foramen in order to minimize the extrusion of debris. Selecting a working length at the level of the apical foramen has been found to result in an increased extrusion of debris through the apex [28].

Reduced instrumentation time has clinical implications, as longer procedures have been shown to potentially increase postoperative discomfort [16]. The observed similarity in the extrusion of debris between the groups suggests that the use of faster preparation techniques, such as PTU, could provide potential benefits to patients without increasing the risk of inflammation. Htun et al. [9] also reported that the ProTaper Next instruments demonstrated a shorter working time compared to the GF instruments, which aligns with our own findings. Similar to the findings of this study, a review of the literature supports the conclusion that rotary NiTi instrumentation requires less working time when compared to manual instrumentation. However, no distinction was observed in relation to cleanliness [29]. Further clinical studies are needed to investigate potential correlations between instrumentation time and postoperative outcomes.

### 4.1. Limitations

This study had some limitations. The study had an in vitro design, and it is not possible to completely simulate the clinical setting in vitro. For instance, the physical pressure of periodontal tissue can prevent the extrusion of debris, which was not simulated in this study [30]. A sponge-like material has been suggested to simulate periapical pressure [31]. Nonetheless, due to the possibility of debris and irrigant absorption, it was not used in this study. Therefore, it is not possible to completely generalize the results to the clinical setting.

### 4.2. Clinical Implications and Future Perspectives

Reducing postoperative pain is one the most important goals of a clinician. Reduced debris extrusion and preparation time can result in postoperative pain reduction [2, 3, 16]. The result of this study might help clinicians to achieve this goal.

Further investigations are required to assess the effect of type and load of bacteria adhering to the extruded debris and the host response on the severity of postoperative pain and edema. Also, clinical trials are required on postoperative pain following the use of different single-file rotary and reciprocating systems. Apical extrusion of debris in curved canals should be investigated in future studies as well.

## 5. Conclusion

In conclusion, GF did not exhibit significantly reduced apical debris extrusion or faster instrumentation compared to PTU in this study. Both rotary systems are faster than manual instrumentation. While efficiency favors contemporary nickel-titanium rotary systems, debris extrusion remains an inherent part of canal instrumentation, necessitating further innovation.

## Figures and Tables

**Table 1:** Kruskal-Wallis test results of the amount of debris extrusion (milligrams) in the study groups.

Grouping variable	*N*	Mean rank^∗∗^	Test statistics		
			Chi-square	Df	*P* value^∗^
ProTaper	22	28.70	2.199	2	0.333
Gentlefile	22	36.98			
Manual	22	34.82			
Total	66				

^∗^Significance level is 0.05. ^∗∗^Milligrams.

**Table 2:** Mean and standard deviation of time (second) in the studied groups.

Grouping variable	*N*	Mean	Std. deviation	95% confidence interval for mean		Minimum	Maximum	*P* value^∗^
				Lower bound	Upper bound			
ProTaper	22	142.36	18.49	134.17	150.56	173.00	110.00	<.001
Gentlefile	22	166.82	39.90	149.13	184.51	233.00	96.00	
Manual	22	232.27	52.83	208.85	255.70	345.00	145.00	
Total	66	180.48	54.67	167.045	193.93	345.00	96.00	

^∗^One-way ANOVA.

## Data Availability

The data used to support the findings of this study were supplied by corresponding author under license, and data will be available on request. Requests for access to these data should be made to corresponding author before 12 months from publication.
